# Interplay with the Mre11-Rad50-Nbs1 complex and phosphorylation by GSK3β implicate human B-Myb in DNA-damage signaling

**DOI:** 10.1038/srep41663

**Published:** 2017-01-27

**Authors:** Sarah Marie Henrich, Clemens Usadel, Eugen Werwein, Kamila Burdova, Pavel Janscak, Stefano Ferrari, Daniel Hess, Karl-Heinz Klempnauer

**Affiliations:** 1 Institut for Biochemistry, Westfälische-Wilhelms-Universität, D-48149 Münster, Germany; 2 Graduate School of Chemistry (GSC-MS), Westfälische-Wilhelms-Universität, D-48149 Münster, Germany; 3 Institute of Molecular Genetics, Academy of Sciences of the Czech Republic, 143 00 Prague, Czech Republic; 4 Institute of Molecular Cancer Research, University of Zurich, Winterthurerstr.190, CH-8057 Zürich, Switzerland; 5 Friedrich Miescher Institute for Biomedical Research, Maulbeerstr. 66, CH-4058 Basel, Switzerland

## Abstract

B-Myb, a highly conserved member of the Myb transcription factor family, is expressed ubiquitously in proliferating cells and controls the cell cycle dependent transcription of G2/M-phase genes. Deregulation of B-Myb has been implicated in oncogenesis and loss of genomic stability. We have identified B-Myb as a novel interaction partner of the Mre11-Rad50-Nbs1 (MRN) complex, a key player in the repair of DNA double strand breaks. We show that B-Myb directly interacts with the Nbs1 subunit of the MRN complex and is recruited transiently to DNA-damage sites. In response to DNA-damage B-Myb is phosphorylated by protein kinase GSK3β and released from the MRN complex. A B-Myb mutant that cannot be phosphorylated by GSK3β disturbs the regulation of pro-mitotic B-Myb target genes and leads to inappropriate mitotic entry in response to DNA-damage. Overall, our work suggests a novel function of B-Myb in the cellular DNA-damage signalling.

B-Myb is a highly conserved member of the Myb proto-oncogene family that is ubiquitously expressed in proliferating cells and performs essential roles as transcription factor^1^. Studies of mammalian B-Myb and its *Drosophila melanogaster* homolog have identified B-Myb as key interaction partner of the evolutionarily conserved Myb-MuvB/DREAM multiprotein complex that regulates the transcription of specific target genes in a cell cycle dependent manner^2^,^3^. The composition of the Myb-MuvB/DREAM complex varies during the cell cycle. In resting cells, the DREAM complex consists of E2F4 and either p130 or p107 and the MuvB core (which is formed by Lin-9, Lin-37, Lin-54, Lin-5 and RBBP4) and acts as a repressor of E2F target genes. In S-phase, the MuvB core complex associates with B-Myb, which then targets it to the promoters of genes required for the G2/M transition and mitosis, thereby activating their transcription^4^,^5^,^6^,^7^,^8^,^9^,^10^. In addition, B-Myb activity itself is highly regulated during the cell cycle by transcriptional and post-transcriptional mechanisms. Notably, phosphorylation of B-Myb by Cyclin A/Cdk2 at the onset of S-phase stimulates its transactivation potential by relieving repressive effects exerted by its C-terminal domain and also triggers its degradation by the ubiquitin-dependent Cdc34-SCF^p45Skp2^ pathway^11^,^12^,^13^,^14^,^15^,^16^,^17^. B-Myb has been shown to interact with several other proteins in addition to the MuvB complex, including cyclin D1^18^,^19^, poly-(ADP-ribose) polymerase (PARP)^20^, nucleolin^21^, p300^19^,^22^, TAFII250^23^ and N-CoR/SMRT^24^.

Recent evidence has suggested that the role of B-Myb as a cell cycle regulated transcription factor is only one aspect of its function in proliferating cells. During mitosis, B-Myb interacts with clathrin and filamin to form the so-called Myb-Clafi complex, which has been implicated in mitotic spindle formation^25^, emphasizing that B-Myb has also “non-transcriptional” roles. Recently, it was shown that B-Myb stimulates G1/S transition independently of its sequence-specific DNA-binding activity and affects the DNA-replication program, further highlighting the complex manner of cell cycle regulation by B-Myb^26^,^27^.

Several findings have suggested that B-Myb is also involved in the DNA-damage response. Knock-out of B-Myb in chicken DT40 cells decreases the survival of the cells after treatment with DNA damaging agents^28^. Consistent with this, B-Myb is required for the recovery from a DNA-damage induced cell cycle arrest^29^. More recently, we have observed that UV irradiation leads to a switch from Cyclin/Cdk-dependent to Jnk- and p38 kinase-dependent phosphorylation of B-Myb, thereby changing the phosphorylation status of B-Myb^30^. However, the exact role of B-Myb in the DNA-damage response has not been revealed by these studies.

We have discovered that B-Myb directly interacts with the Mre11-Rad50-Nbs1 (MRN) complex, a key player in the response to DNA double strand breaks (DSBs)^31^,^32^. We show that B-Myb is transiently recruited to sites of DNA-damage and is phosphorylated in a GSK3β-dependent manner. Our work implicates B-Myb for the first time in the cellular response to DNA DSBs.

## Results

### B-Myb is associated with the MRN complex

**Figure 1:**
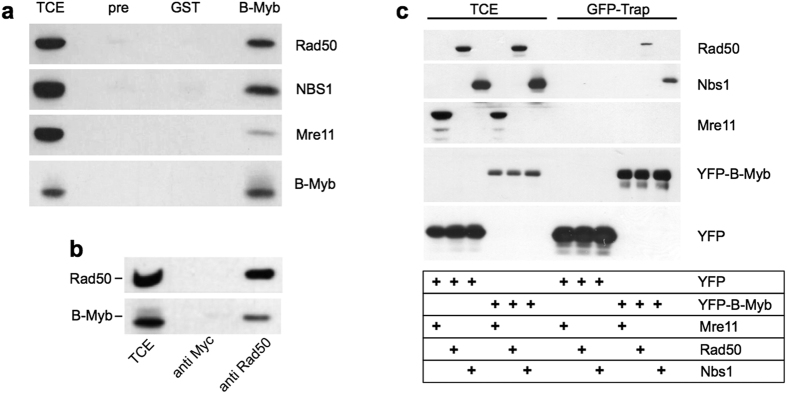
B-Myb is associated with the MRN complex. (**a**) Extracts from untreated MCF7 cells were precipitated with antibodies against B-Myb, pre-immune serum (pre) or antiserum against an unrelated protein (GST). Immunoprecipitates and aliquots of the total cell extract (TCE) were immunoblotted using antibodies against Rad50, Nbs1, Mre11 and B-Myb. (**b**) Cell extracts from untreated MCF7 cells were immunoprecipitated with antibodies against Rad50 or the Myc-tag. Immunoprecipitates were analyzed with antibodies against Rad50 and B-Myb. (**c**) QT6 fibroblasts were transfected with expression vectors for YFP, YFP-B-Myb, Mre11, Rad50 and Nbs1. Cell extracts were incubated with GFP-trap beads (containing a GFP-binding protein) and bound proteins (GFP-trap) as well as aliquots of the total cell extracts (TCE) were analyzed by western blotting with antibodies against Rad50, Nbs1, Mre11 and YFP.

To explore the role of B-Myb in DNA-damaged cells we sought to identify B-Myb interaction partners with known functions in the DNA-damage response. Because loss of B-Myb in mouse ES-cells causes accumulation of DNA DSBs^27^ we tested if B-Myb associates with the MRN complex, a key player in the repair of DNA DSBs that is recruited to the sites of DSBs (Carney *et al*.^33^; Tauchi *et al*.^34^). Immunoprecipitation of B-Myb from extracts of MCF7 cells grown under normal conditions showed that all three components of the MRN complex, Mre11, Rad50 and Nbs1, were co-precipitated with an antiserum against the B-Myb DNA-binding domain (Fig. 1a). Control experiments with an unrelated antiserum or pre-immune serum confirmed the specificity of the co-precipitation. Co-precipitation of Mre11, Rad50 and Nbs1 was also observed when an antiserum against the C-terminal region of B-Myb or another cell-line was used (Supplementary Fig. 1). Furthermore, the interaction was confirmed by co-precipitation of B-Myb with antibodies against Rad50 (Fig. 1b). Interaction of B-Myb with the MRN complex was also confirmed by co-precipitation in the presence of ethidium bromide (Supplementary Fig. 1), suggesting that the interaction is not due to contaminating DNA. Because the experiments were performed with extracts from cells grown under non DNA-damaging conditions these results indicated that B-Myb interacts with the MRN complex in the absence of DNA-damage.

To identify the primary B-Myb interaction partner in the MRN complex, we transfected fibroblasts with expression vectors for a YFP/B-Myb fusion protein and individual MRN complex members and analyzed the cell extracts using GFP-trap beads, which contain a covalently bound high-affinity GFP-binding protein. As control, a YFP expression vector was used instead of the YFP/B-Myb vector. Figure 1c shows that Rad50 and Nbs1 but not Mre11 were able to interact with the YFP/B-Myb fusion protein, indicating that B-Myb independently interacts with Rad50 and Nbs1.

### B-Myb is recruited to sites of DNA-damage

**Figure 2:**
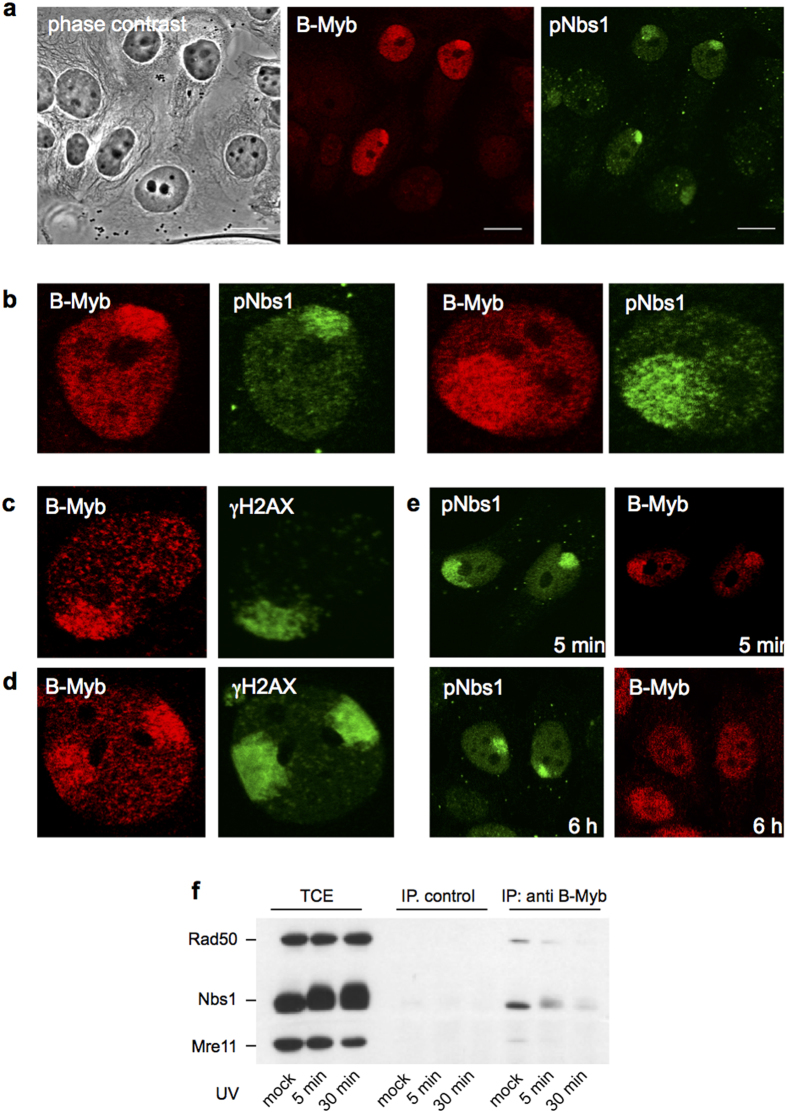
B-Myb is recruited to sites of DNA double strand breaks. UV-microirradiated MCF7 (**a–c**) and HepG2 (**d,e**) cells were analyzed by immunofluorescence microscopy with antibodies against B-Myb, phosphorylated Nbs1(Ser343) and γH2AX. (**f**) Extracts from untreated or UV-irradiated (30 J/m2) BrdU-prelabeled MCF7 cells were precipitated with antibodies against B-Myb or pre-immune serum (control). Immunoprecipitates and aliquots of the total cell extract (TCE) were immunoblotted using antibodies against Rad50, Nbs1, Mre11 and B-Myb.

To investigate if B-Myb is recruited to DNA-damage sites we micro-irradiated BrdU-labeled cells through porous polycarbonate filters, as described by Suzuki *et al*.^35^. MCF7 cells were cultured for 72 h in the presence of 10 μM BrdU and then irradiated with UVC light. We validated the induction of DSBs under these conditions by staining with antibodies against known DNA-damage associated proteins or post-translational modifications (Supplementary Fig. 2). We then stained the micro-irradiated cells with a B-Myb specific monoclonal antibody and phospho-specific antibodies against Nbs1 (pNbs1Ser343) as a marker of DSBs. As shown in Fig. 2a and b, B-Myb clearly localized with phosphorylated Nbs1 to the irradiated area, indicating that it accumulated at regions of DSBs. Importantly, the majority of cells that were positive for B-Myb staining (i.e. presumably S- or G2-phase cells) showed localization of B-Myb with γH2AX or pNbs1 in the irradiated area. B-Myb was also enriched in regions positive for γH2AX in MCF7 (Fig. 2c) and HepG2 cells (Fig. 2d), indicating that the recruitment of B-Myb to DNA-damage regions was not cell line-specific. Finally, we analyzed the cells at different times after irradiation (Fig. 2e). B-Myb foci appeared rapidly within 5 minutes after irradiation but disappeared after longer times although the phospho-Nbs1 staining persisted. Overall these experiments showed that B-Myb is recruited rapidly and transiently to chromatin regions that contain DSBs.

Recruitment of B-Myb to sites of DNA-damage was also observed, although less prominently, when the cells were not pre-incubated with BrdU (Supplementary Fig. 3). Figure 2 shows that only a part of B-Myb was recruited to the DNA-damage site while a fraction of B-Myb remained distributed throughout the nucleus. The detection of B-Myb recruitment to DNA-damage sites therefore critically depends on the background of the diffusely distributed fraction of B-Myb and requires a sufficient amount of DNA-damage. This is achieved by pre-labeling the cells with BrdU, which amplifies the damaging effect of the irradiation. We also performed extraction experiments with detergent-containing buffers, however, this did not improve the visualization of the DNA-damage associated B-Myb, presumably because the DNA-damage associated B-Myb does not bind stronger to chromatin than the bulk of B-Myb. We also induced DSBs with ionizing radiation (IR) up to 20 Gy but were not able to detect recruitment of B-Myb to IR-induced γH2AX foci. Because such foci contain only one or a few DSBs^36^ the detection of any recruitment of B-Myb is probably obscured by the diffusely distributed fraction of B-Myb.

We have previously reported that UV-irradiation or heat stress induces association of a modified form of B-Myb phosphorylated at Thr-487 with so-called SC35 nuclear speckles^30^. The UV-induced SC35 speckle association of B-Myb described previously is clearly distinct from the immediate recruitment of B-Myb to sites of DNA-damage shown in Fig. 2 and appears to be a more general stress response. Importantly, the SC35 speckles are not the sites of DNA-damage and the association of phospho-B-Myb(T487) with SC35 speckles did not occur within minutes after the UV-irradiation but developed over a period of several hours.

### B-Myb dissociates from the MRN complex after induction of DNA-damage

Next, we were interested to see if the transient nature of the recruitment of B-Myb to γH2AX foci was due to a dissociation of B-Myb from the MRN complex. To provide conditions comparable to the microirradiation experiments we used MCF7 cells that had been pre-labeled with BrdU before UV-treatment. Extracts from UV-irradiated MCF7 cells were then analyzed by co-immunoprecipitation for interaction of B-Myb with the MRN complex. Figure 2f shows that the B-Myb/MRN interaction was already reduced 5 minutes after UV-irradiation and became very weak after longer times, indicating that the DNA-damage dissociates B-Myb from the MRN complex.

### Mapping the domains that mediate the interaction of Nbs1 and B-Myb

**Figure 3:**
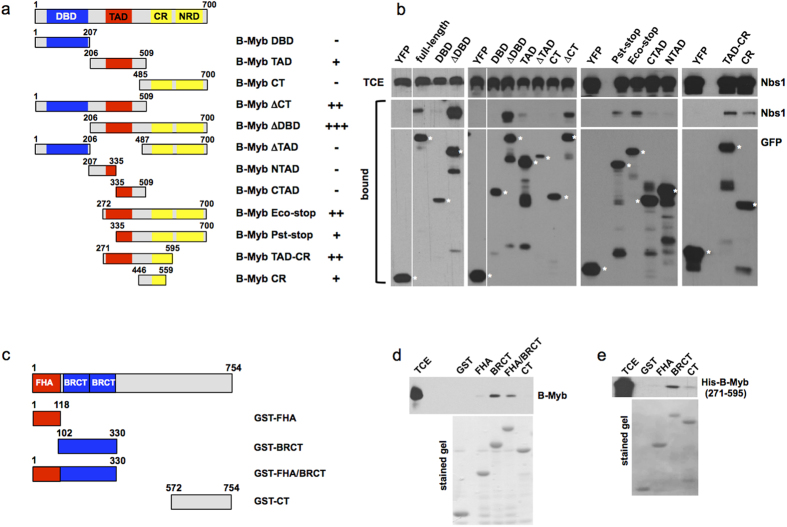
Mapping the binding site involved in the B-Myb/Nbs1 interaction. (**a**) Schematic illustration of the domains of B-Myb (DBD: DNA binding domain; TAD: transactivation domain; CR: conserved region; NRD: negative regulatory domain) and GFP/B-Myb deletion constructs. The numbers refer to amino acids. (**b**) QT6 fibroblasts were transfected with expression vectors for Nbs1 and the indicated GFP/B-Myb constructs. Cell extracts were incubated with GFP-trap beads and the bound proteins were analyzed by western blotting with antibodies against Nbs1 or GFP (middle and bottom panels). White asterisks mark full-length GFP/B-Myb proteins. In addition, aliquots of total cell extracts were analyzed with Nbs1 antibodies (top panels). (**c**) Schematic illustration of the domains of Nbs1 and GST-Nbs1 fusion proteins used for binding experiments. The numbers refer to amino acids. (**d**) Glutathione-sepharose beads loaded with the indicated GST fusion proteins were incubated with extract from QT6 fibroblasts transfected with B-Myb expression vector. Bound proteins and aliquots of the cell extracts were analyzed by western blotting using antibodies against B-Myb. Panel e shows a similar binding experiment in which bacterially expressed His-tagged B-Myb (consisting of amino acids 271 to 595 of B-Myb) was used instead of cell extract. The bottom panels in d and e show coomassie brilliant blue-stained gels to confirm equal loading of the beads with GST proteins.

In the MRN complex, Nbs1 acts as a scaffold that recruits other proteins to DSBs, for example CtIP and MDC1^37^,^38^. We therefore decided to characterize the interaction of B-Myb and Nbs1 in more detail. To identify the domains of B-Myb that mediate this interaction we analyzed the binding of Nbs1 to a set of truncated GFP-B-Myb fusion proteins (Fig. 3a). As illustrated in Fig. 3b, the B-Myb DNA-binding domain was unable to bind to Nbs1 whereas a B-Myb construct lacking the DNA-binding domain bound Nbs1 very efficiently. This supports our earlier conclusion that the co-precipitation of B-Myb and Nbs1 is not due to unspecific binding of both proteins to DNA. Figure 3b also shows that a C-terminally deleted construct lacking the negative regulatory domain (NRD) was able to bind Nbs1. A construct derived from the center of B-Myb, which harbors the transactivation domain (B-Myb-TAD), bound Nbs1 weakly while splitting this construct in two halves (B-Myb-NTAD and -CTAD) resulted in loss of binding activity. This indicated that the middle part of B-Myb is involved in binding Nbs1. Finally we were able to map the binding region to a small B-Myb construct (B-Myb CR, amino acid residues 446–559) that showed significant binding to Nbs1. However, it is also apparent that the smaller B-Myb constructs bound Nbs1 less efficiently than the longer constructs. This suggests that either the correct folding of the smaller constructs into their native conformation is impaired or that Nbs1 binds to a composite site formed by different parts of B-Myb. Because of this we were not able to define the Nbs1 binding site in B-Myb more precisely.

To map the binding region for B-Myb within Nbs1 we used bacterially expressed GST-Nbs1 proteins that encompass different parts of Nbs1 (Fig. 3c). *In vitro* pull-down experiments with extract from cells transfected with a B-Myb expression vector identified the tandem BRCT domain as the binding site for B-Myb (Fig. 3d).

To investigate whether B-Myb binds directly to Nbs1 we expressed the central part of B-Myb as a His-tagged protein in bacteria, purified it and examined its interaction with bacterially expressed GST-Nbs1. Figure 3e shows that His-tagged B-Myb binds to the tandem BRCT domain of Nbs1, demonstrating that the interaction occurs in the absence of other eukaryotic proteins. Taken together, we have identified a binding region in the central part of B-Myb that interacts directly with the tandem BRCT domain of Nbs1.

### B-Myb is not essential for DNA repair

To further investigate the role of B-Myb in DNA-damaged cells we compared the repair of the DNA-damage in cells expressing normal or reduced levels of B-Myb. We used previously described eGFP-based repair assays for homologous recombination (HR) and non-homologous end-joining (NHEJ)^39^,^40^. As shown in Supplementary Fig. 4 there was no significant effect of the B-Myb knock-down on the repair efficiency in both assays. We also performed comet assays to quantify DNA-damage at the single cell level (Supplementary Fig. 5). In this assay cells are embedded in agarose gels, lysed *in situ* and subjected to electrophoresis. After staining with propidium iodide damaged DNA is visible as a tail originating from the cell nucleus. These experiments showed that, there was no significant difference between B-Myb knock-down and control cells in the amount of DNA-damage induced by UV-irradiation or etoposide. We therefore concluded that B-Myb does not play essential or major roles in the repair of damaged DNA.

### UV-irradiation induces a phosphorylated form of B-Myb

**Figure 4:**
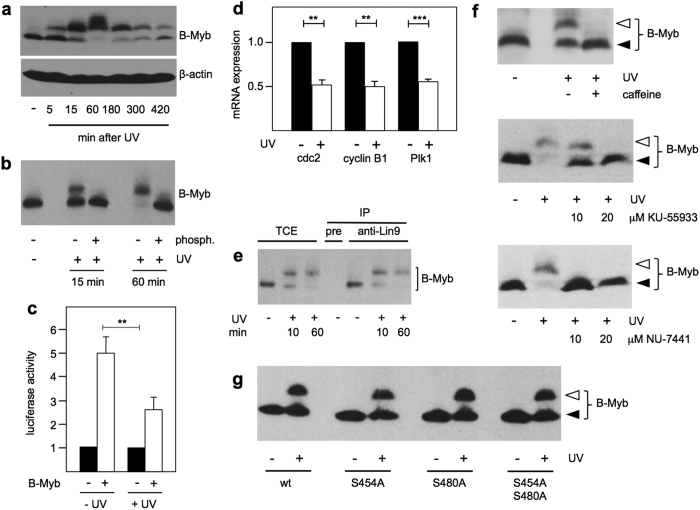
UV-irradiation induces phosphorylation of B-Myb. (**a**) HepG2 cells were prelabeled for 72 hours with BrdU, UV irradiated (30 J/m2) and harvested after the indicated times. Un-irradiated cells served as control. Total cell extracts were analyzed by western blotting with antibodies against B-Myb and β-actin. (**b**) B-Myb was immunoprecipitated from extracts of HepG2 cells prelabeled with BrdU, UV-irradiated and further incubated for 15 or 60 min. Immunoprecipitates were subsequently incubated with alkaline phosphatase for 1 h at 37 °C. Samples lacking phosphatase were incubated in parallel. (**c**) Hela cells were transfected with the Myb-dependent reporter gene pGL4-5xMRE(GG)-myc, the β-galactosidase plasmid pCMVb and expression vector for human B-Myb, as indicated. The cells were prelabeled with BrdU and UV-irradiated, as in A. The bars show the luciferase activity, normalized against b-galactosidase activity, of cells harvested 4 hr after UV-irradiation. (**d**) Hek293 cells were prelabeled with BrdU and UV irradiated as in A. RNA was isolated after 4 hr and analyzed by real-time PCR for the expression of known B-Myb target genes. mRNA expression levels were normalized to β-actin and expression in unirradiated cells was set to 1. (**e**) HepG2 cells were prelabeled for 72 hours with BrdU and UV irradiated (30 J/m2). Control cells were not irradiated. Cell extracts were immunoprecipitated with preimmune serum (pre) or antiserum against Lin9 and analyzed by SDS-PAGE and western blotting with B-Myb antiserum. Aliquots of the total cell extracts (TCE) were analyzed in parallel. (**f**) HepG2 cells were UV-irradiated in the absence or presence of 12 mM caffeine or the indicated concentrations of KU-55933 or NU7441. The cells were harvested after 30 min and analyzed by western blotting with antiserum against B-Myb. Unirradiated cells served as control. (**g**) GFP/B-Myb(TAD) wild-type and mutant proteins were expressed in Hela cells prelabeled for 72 hours with BrdU and UV irradiated. The cells were harvested after 60 min and total cell extracts were analyzed by western blotting with antibodies against GFP.

Western blotting of B-Myb after UV-irradiation of BrdU-labeled cells revealed a substantial electrophoretic mobility shift, which was already visible 5 min after induction of DNA-damage, became very prominent after 60 min and decreased again, but was still visible 7 hours after UV-irradiation (Fig. 4a). Figure 4b shows that the mobility shift was completely abolished by phosphatase treatment, indicating that it was due to phosphorylation. Similar UV-induced mobility shifts were observed in different cell lines (Supplementary Fig. 6).

Because our data suggested that B-Myb is not directly involved in the repair of the DNA-damage we speculated that the DNA-damage-induced phosphorylation of B-Myb might play a role in downstream DNA-damage signalling to B-Myb target genes. As a first step to address this possibility we compared the activity of B-Myb before and after UV-irradiation in a luciferase reporter experiment and found that the transactivation potential of B-Myb was decreased by UV (Fig. 4c). Consistent with this decrease the expression of several authentic B-Myb target genes was decreased after UV-irradiation (Fig. 4d). Finally, we asked if the phosphorylated form of B-Myb is incorporated into the MuvB complex. We immunoprecipitated extracts from UV-irradiated and control cells with antibodies against Lin9, the direct interaction partner of B-Myb within the MuvB complex. Figure 4e shows that the UV-induced phosphorylated form of B-Myb was co-precipitated via Lin9, suggesting that it was associated with the MuvB complex. Together, these findings support a role of B-Myb in downstream DNA-damage signalling.

### UV-induced phosphorylation of B-Myb is mediated by protein kinase GSK3β

The protein kinases ATM and DNA-PK play key roles in the repair of DSBs by HR or NHEJ, respectively. To address the involvement of these kinases in the phosphorylation of B-Myb we irradiated cells in the absence or presence of inhibitors of these kinases. As shown in Fig. 4f caffeine, the ATM-inhibitor KU-55933 and the DNA-PK inhibitor NU-7441 clearly inhibited the appearance of the slower migrating form of B-Myb, suggesting that these kinases were directly or indirectly involved in the phosphorylation of B-Myb. Human B-Myb contains only two S/TQ-motifs located at amino acid positions 454 and 480, which fulfill the recognition requirement for phosphorylation by ATM or DNA-PK. Surprisingly, mutation of both motifs had no effect on the UV-induced phosphorylation (Fig. 4g), suggesting that a protein kinase acting downstream of ATM or DNA-PK is involved. The checkpoint kinases Chk1 and Chk2 are established downstream kinases that mediate many effects of ATM kinase. However, the Chk1 and Chk2 inhibitor AZD-7762 failed to inhibit the phosphorylation of B-Myb, indicating that none of these kinases are involved. We also tested inhibitors for p38, JNK and cyclin-dependent kinases, but none of them inhibited the phosphorylation of B-Myb.

**Figure 5:**
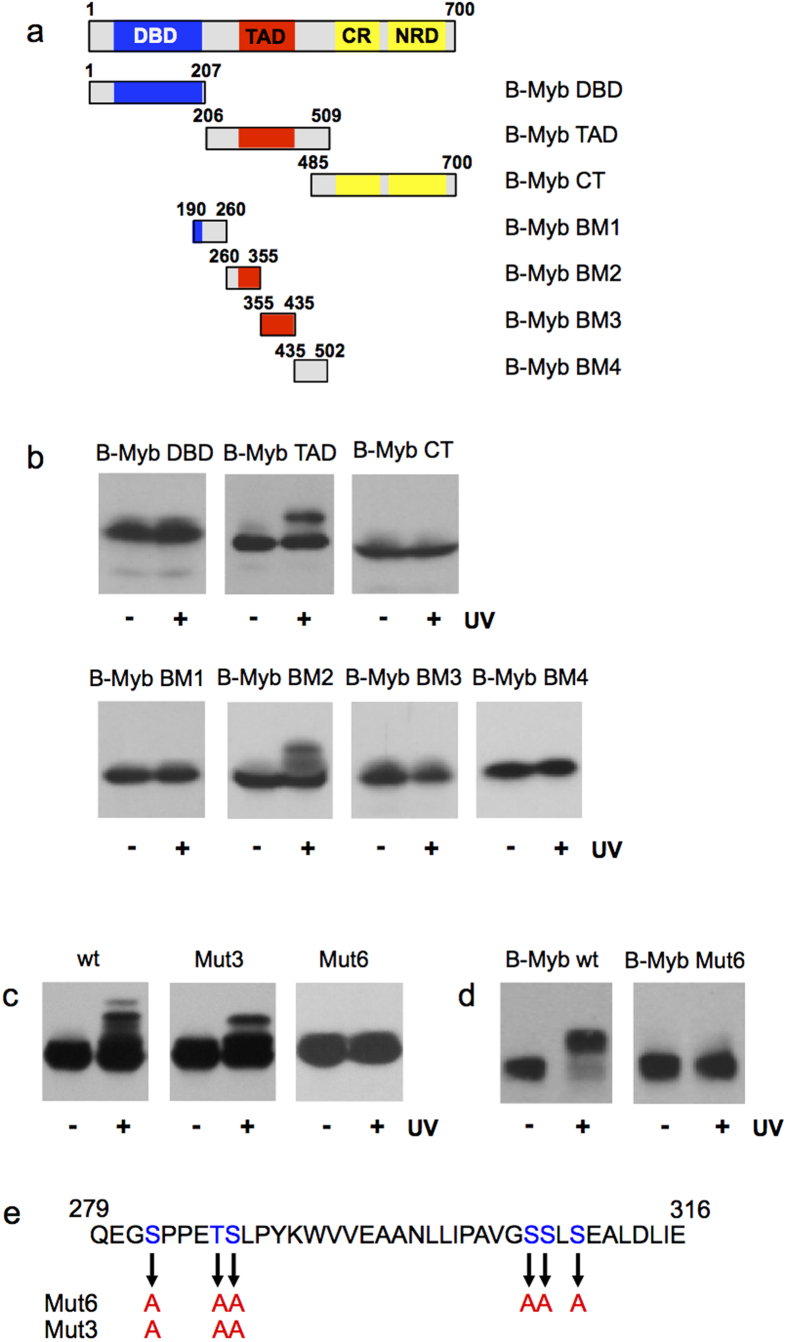
Mapping of the sites of UV-induced phosphorylation of B-Myb. (**a**) GFP/B-Myb constructs are shown schematically at the top. (**b**) The indicated B-Myb constructs were expressed in QT6 fibroblasts prelabeled with BrdU. The cells were UV irradiated (30 J/m2) and harvested after 60 min. Unirradiated cells served as controls. Total cell extracts were analyzed by western blotting with antibodies against GFP (bottom panels). (**c**) Wild-type B-Myb BM2 and mutants Mut3 and Mut6 were analyzed as in panel B. (**d**) Full-length B-Myb-wt and B-Myb-Mut6 were analyzed as in panel B. (**e**) Overview of amino acid residues changed to Ala in the B-Myb mutants.

To further characterize the UV-induced phosphorylation, we mapped the part of B-Myb that is responsible for the UV-induced mobility shift. Expression of GFP-fusion proteins containing different parts of B-Myb revealed that the sequences responsible for the UV-induced mobility shift lie between amino acids 260 and 355 of B-Myb (Fig. 5a and b). To identify the sites of phosphorylation, we compared the UV-induced lower-mobility form of GFP/B-Myb-ΔDBD by mass spectrometry to the same protein isolated from unirradiated cells. We only found peptides containing phosphorylated Ser-282, Thr-286 and Ser-287 to be enriched in the lower-mobility form, indicating that these residues are phosphorylated in response to UV (Supplementary Fig. 7). To confirm this, we mutated these residues to alanine in the B-Myb-BM2 construct (generating Mut3a), and analyzed the mobility of the mutant protein with and without UV-irradiation. Figure 5c shows that the mobility shift was less pronounced but not abolished, indicating that additional sites are phosphorylated after UV-irradiation. We then mutated additionally a cluster of serine residues at positions 306, 307 and 309. In the resulting protein (Mut6) the UV-induced mobility shift was completely abolished, indicating that all or some of these aminoacids are also phosphorylated. When all of these mutations were introduced into full-length B-Myb the resulting protein (B-Myb-Mut6) no longer showed a UV-induced mobility shift (Fig. 5d).

**Figure 6:**
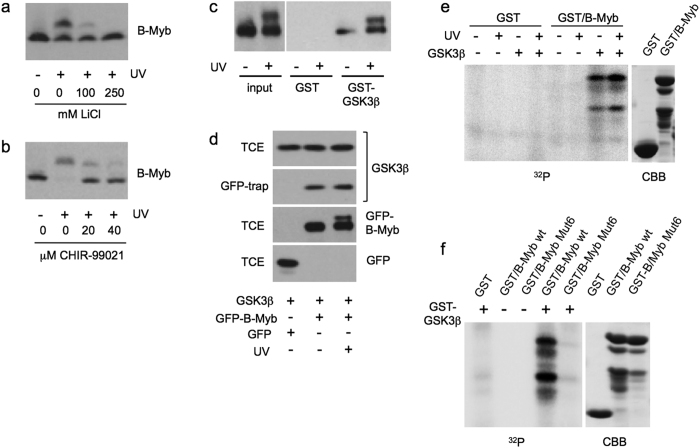
UV-induced phosphorylation of B-Myb is dependent on protein kinase GSK3β. (**a,b**) HepG2 cells were prelabeled for 72 hours with BrdU and UV irradiated in the absence or presence of lithium chloride or CHIR-99021. The cells were harvested after 60 min and analyzed by western blotting with antibodies against B-Myb. Unirradiated cells served as control. (**c**) Glutathione-sepharose beads loaded with GST or GST-GSK3β were incubated with extract from un-irradiated or UV-irradiated QT6 cells that had been transfected with a B-Myb expression vector. Bound proteins and aliquots of the cell extracts were analyzed by western blotting using antibodies against B-Myb. (**d**) QT6 fibroblasts were transfected with expression vectors for GFP, GFP-B-Myb and HA-tagged GSK3β, as indicated. UV irradiation was performed as in panel a. Cell extracts were incubated with GFP-trap beads and bound proteins (GFP-trap) as well as aliquots of the total cell extracts (TCE) were analyzed by western blotting with antibodies against the HA-tag and GFP. (**e**) *In vitro* protein kinase assay. Sepharose beads loaded with GST or GST/B-Myb(260–355) were incubated *in vitro* with γ32P-ATP and GSK3β immunoprecipitated from UV-irradiated or un-irradiated cells expressing HA-GSK3β. Radiolabeled proteins were visualized by autoradiography. A coomassie brilliant blue-stained gel of the GST-proteins is shown on the right. (**f**) *In vitro* protein kinase assay using bacterially expressed GST-GSK3β. The indicated GST/B-Myb proteins were analyzed by SDS-PAGE and staining with coomassie brilliant blue (right panel) or were incubated together with GST-GSK3β and γ32P-ATP, followed by SDS-PAGE and autoradiography (left panel).

Recent work has implicated the protein kinase GSK3β in the phosphorylation and subsequent proteasomal degradation of cyclin D1 in response to genotoxic stress^41^. Because the sequence at Ser-282, Thr-286 and Ser-287 resembles the consensus GSK3β phosphorylation motif S/TXXXS/T(p), we examined the effects of the GSK3β inhibitors LiCl and CHIR-99021 on the UV-induced phosphorylation of B-Myb. As shown in Fig. 6a and b, both inhibitors clearly suppressed the UV-induced mobility shift of B-Myb, implicating GSK3β in the phosphorylation of B-Myb.

To obtain evidence for a direct role of GSK3β in the phosphorylation of B-Myb we investigated if both proteins interact. We performed a GST pull-down experiment using sepharose beads loaded with GST or GST-GSK3β (Fig. 6c). Clearly, B-Myb was bound to GST-GSK3β, indicating that B-Myb and GSK3β interact *in vitro*. We also transfected cells with expression vectors for GFP-B-Myb and GSK3β and analyzed the cell extracts using GFP-trap beads. Figure 6d shows that GSK3β was co-precipitated with GFP-B-Myb but not with GFP, confirming that GSK3β binds to B-Myb also *in vivo*. Interestingly, we did not observe an increase of the interaction after UV irradiation, indicating that the stimulation of the phosphorylation by UV was not due to increased binding of B-Myb and GSK3β.

Finally, we performed *in vitro* protein kinase assays with a bacterially expressed B-Myb protein and GSK3β immunoprecipitated from extracts of transfected cells. This experiment showed that GSK3β was able to phosphorylate GST/B-Myb *in vitro* (Fig. 6e). To exclude that an unknown protein kinase was co-precipitated with GSK3β we subjected bacterially expressed B-Myb-BM2 to an *in vitro* kinase assay with bacterially expressed and purified GSK3β (Fig. 6f). This demonstrated that wild-type B-Myb, but not the Mut6 version, was phosphorylated by purified GSK3β. Taken together, these data indicate that GSK3β is the protein kinase responsible for the UV-induced phosphorylation of B-Myb.

### Disruption of the B-Myb/Nbs1 interaction requires ATM activity

**Figure 7:**
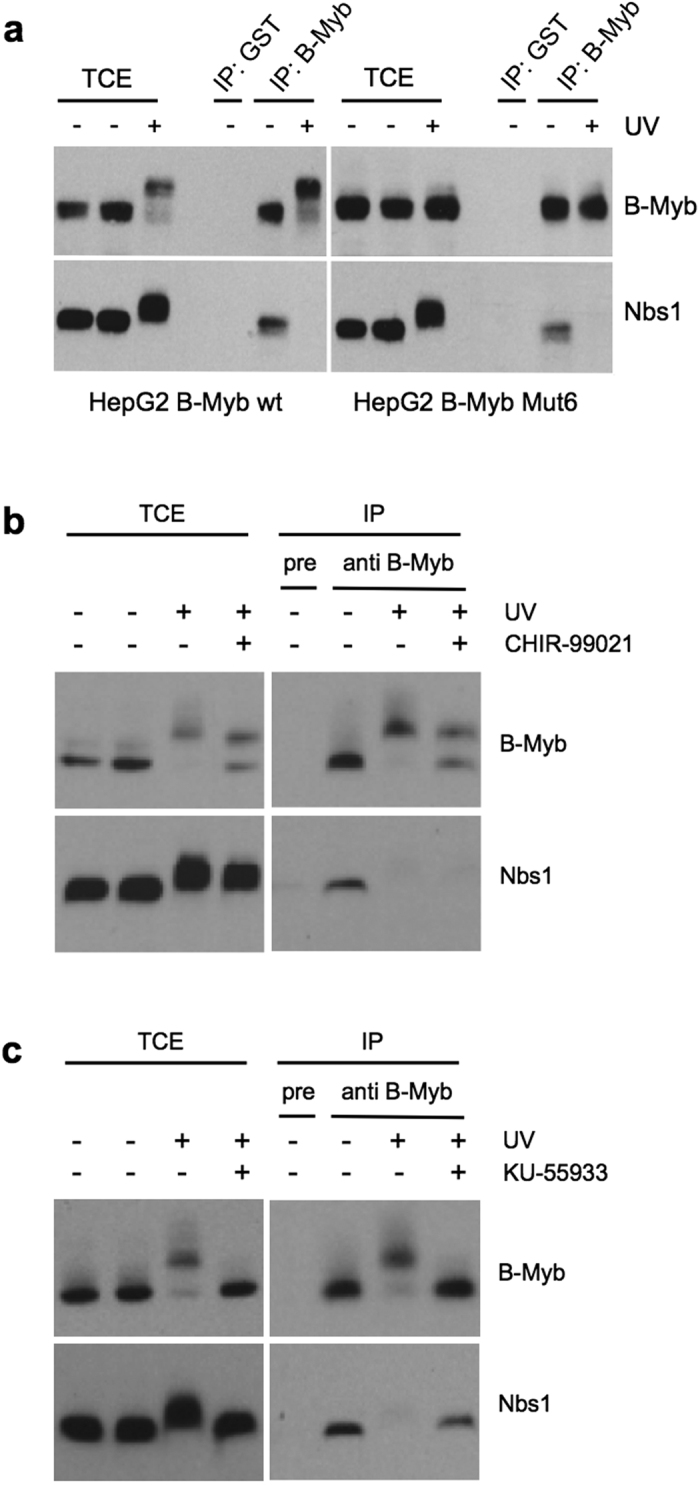
UV-induced phosphorylation disrupts the B-Myb/Nbs1 interaction. (**a**) HepG2 cells infected with lentiviruses encoding wild-type B-Myb or B-Myb-Mut6 were prelabeled for 72 hours with BrdU and UV irradiated (30 J/m2). Control cells were not irradiated. The cells were harvested after 60 min and analyzed by immunoprecipitation with B-Myb specific or control antiserum. Immunoprecipitates and aliquots of the total cell extracts were analyzed by western blotting with antibodies against B-Myb or Nbs1. (**b,c**) Un-infected HepG2 cells were prelabeled with BrdU and UV irradiated as in a. in the presence or absence of 20 μM CHIR-99021 (**b**) or 50 μM KU-55933 (**c**). The cells were analyzed as described in a.

Because the association of B-Myb and Nbs1 was abolished upon induction of DNA-damage (Fig. 2f) we were interested to know whether the GSK3β-induced phosphorylation of B-Myb triggers the disruption of the interaction between both proteins. To address this, we expressed full-length B-Myb-Mut6 (lacking the GSK3β phosphorylation sites) as well as wild-type B-Myb in HepG2 cells, using lentiviral infection, and subjected extracts from these cells to co-immunoprecipitation analysis. As shown in Fig. 7a, induction of DNA-damage abolished the B-Myb/Nbs1 interaction even when B-Myb was not phosphorylated by GSK3β. Similarly, treatment of HepG2 cells with the GSK3β inhibitor CHIR-99021 did not prevent the DNA-damage-induced disruption of the interaction of endogenous B-Myb and Nbs1 (Fig. 7b). These data indicated that the disruption of the B-Myb/Nbs1 interaction is independent of the GSK3β-induced phosphorylation. By contrast, when HepG2 cells were treated with the ATM-inhibitor KU-55933, Nbs1 was coprecipitated via B-Myb even after induction of DNA-damage (Fig. 7c), demonstrating that the release of B-Myb from Nbs1 requires ATM kinase activity. A possible interpretation of these results is that ATM-dependent phosphorylation of the MRN complex, whose subunits are all phosphorylated by ATM^42^,^43^, rather than the phosphorylation of B-Myb by GSK3β is the primary trigger to release B-Myb from Nbs1. Since ATM is activated at the sites of DSBs this also suggests that B-Myb is released from the MRN complex after it was recruited to the DNA-damage site, providing an explanation for the transient nature of B-Myb recruitment to the DNA-damage foci in Fig. 2e.

### A GSK3β non-phosphorylatable B-Myb mutant disturbs expression of pro-mitotic B-Myb target genes and promotes mitosis in the presence of DNA-damage

**Figure 8:**
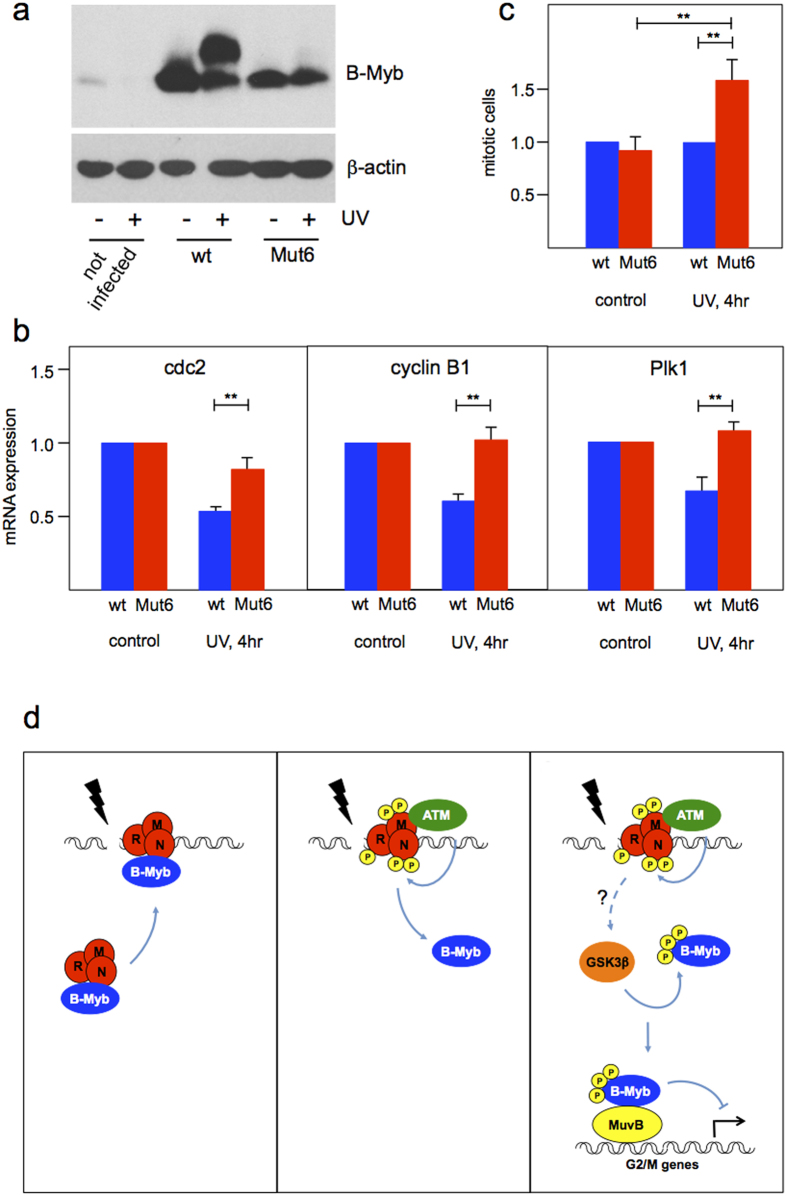
B-Myb phosphorylation by GSK3β is required for the downregulation of pro-mitotic B-Myb target genes and to prevent entry into mitosis after DNA-damage. (**a**) Expression of B-Myb in HepG2 cells infected with lentiviruses encoding wild-type B-Myb and B-Myb-Mut6. B-Myb expression in uninfected cells is shown for comparison. (**b**) Wild-type B-Myb and B-Myb-Mut6 expressing HepG2 cells were prelabeled for 72 hours with BrdU and UV irradiated (30 J/m2), as indicated. RNA was isolated after 4 hr and analyzed by real-time PCR for the expression of pro-mitotic B-Myb target genes. mRNA expression levels were normalized to unirradiated cells. (**c**) Wild-type B-Myb and B-Myb-Mut6 expressing HepG2 cells were treated as in b. Bars show the relative numbers of mitotic cells as determined by staining with antibodies against histone H3 phosphorylated at Ser-10. Numbers for cells expressing mutant B-Myb were normalized to wild-type B-Myb expressing cells treated identically. Asterisks indicate statistical significance (**p < 0.01, Student’s t-test). (**d**) Suggested model for the role of B-Myb in the response to DNA-double strand breaks. B-Myb is recruited via the MRN complex to DNA-damage sites (left). Activation of ATM kinase induces the release of B-Myb from the complex, presumably due to the phosphorylation of the MRN complex (middle). B-Myb is then phosphorylated by GSK3β to dampen the transcription of G2/M genes (right). For further explanations see the text.

To address the role of GSK3β-induced phosphorylation of B-Myb in response to DNA-damage we used HepG2 cells infected with lentiviruses encoding wt B-Myb or the GSK3β non-phosphorylatable mutant B-Myb-Mut6. Figure 8a shows that the expression of the lentivirally encoded B-Myb proteins exceeded that of endogenous B-Myb. Hence, we expected that exogenously expressed mutant B-Myb would override the activity of the endogenous wild-type B-Myb. Since our experiments had shown that the phosphorylated B-Myb associates with Lin9 we examined the expression of known G2/M-phase B-Myb target genes by real-time PCR in cells over-expressing wild-type and mutant B-Myb. Figure 8b shows that the expression of Cyclin B1, Cdc2 and Plk1 was decreased after UV irradiation in cells expressing wild-type B-Myb, whereas DNA-damage induced down-regulation of these genes was impaired in cells expressing B-Myb-Mut6.

To investigate if the increased expression of pro-mitotic B-Myb target genes is accompanied by an increase in mitotic cells we used an antibody against phospho-histone H3 (Ser10) to detect mitotic cells by flow cytometry, Fig. 8c shows that, compared to the control cells expressing wild-type B-Myb, the cell population expressing B-Myb-Mut6 contained an increased proportion of mitotic cells after UV-irradiation.

## Discussion

Two key findings reported here suggest a novel role of B-Myb in the cellular response to DNA double strand breaks: First, B-Myb is physically associated with the MRN complex, a key player in the DNA damage response, that recognizes DSBs and orchestrates the recruitment of other proteins to the DNA damage site^31^,^32^. The B-Myb/MRN association occurs in cells growing under normal conditions but is disrupted by DNA-damage. Second, B-Myb is recruited quickly, i.e. within minutes after induction of DNA damage, and transiently to sites of DSBs. Together these findings suggest that B-Myb is associated with the MRN complex in the absence of DNA damage, is recruited by the MRN complex to the DNA damage site to be subsequently released from the complex. We showed that the ATM-inhibitor KU-55933 prevents the dissociation of B-Myb from the MRN complex (Fig. 7c), indicating that ATM-dependent phosphorylation, possibly of Nbs1 or the other subunits of the MRN complex, disrupts the interaction of B-Myb with the MRN complex. Since ATM kinase is activated by the MRN complex at the DNA damage site this provides a simple explanation for the transient nature of B-Myb recruitment to these sites.

We have identified the tandem BRCT domain of Nbs1 and the central part of B-Myb as the relevant domains that mediate the interaction of both proteins. B-Myb and Nbs1 bind to each other even when expressed in bacteria, i.e. in the absence of other eukaryotic proteins (Fig. 3e), suggesting a direct protein-protein-interaction between B-Myb and Nbs1. Nbs1 contains two phosphopeptide interaction domains, the FHA- and the tandem BRCT-domains, which recruit other proteins in a phosphorylation-dependent manner to DNA-damage sites, such as the CtBP-interacting protein (CtIP) and the mediator of DNA-damage checkpoint 1 (MDC1) protein^31^,^32^. CtIP binds to the FHA-domain whereas MDC1 interacts simultaneously with the FHA and tandem BRCT domain^37^,^38^. By contrast, binding of B-Myb is mediated only by the tandem BRCT domain and appears to be phosphorylation-independent. Binding of MDC1 to Nbs1 is dependent on the phosphorylation of Ser-Asp-Ser-Asp-motifs of MDC1 by casein kinase 2 (CK2)^44^,^45^,^46^. With the exception of a related motif close to its amino terminus B-Myb does not contain any potential CK2 sites. Furthermore, a bacterially expressed protein containing the central part of B-Myb (amino acids 271–595) and presumably lacking eukaryotic-specific phosphorylations was able to bind to Nbs1 (Fig. 3e). Overall, this indicates that B-Myb interacts with Nbs1 by a mechanism that differs from that of other known Nbs1 binding partners.

B-Myb does not appear to play an essential role in the actual repair of damaged DNA. Rather, its conversion to a phosphorylated form within minutes after induction of DNA-damage points to a role of B-Myb in downstream DNA-damage signaling. We have shown that inhibition of ATM activity prevents the phosphorylation of B-Myb (Fig. 4f), however, our data show that B-Myb is not a direct ATM target. Rather, several lines of evidence implicate protein kinase GSK3β in the phosphorylation of B-Myb: The GSK3β-specific inhibitor CHIR-99021 blocks DNA-damage induced phosphorylation of B-Myb. Furthermore, GSK3β interacts with B-Myb *in vitro* and *in vivo* and phosphorylates B-Myb at relevant sites in an *in vitro* protein kinase assay (Fig. 6). How the DNA-damage signal is transmitted to GSK3β is unknown at present and remains to be addressed in future work. Previous work has shown that GSK3β phosphorylates cyclin D1 in response to DNA-damage, suggesting the existence of a presently unexplored signaling pathway between ATM and GSK3β that might also be responsible for the ATM-dependent phosphorylation of B-Myb by GSK3β^41^. The DNA-dependent protein kinase (DNA-PK) inhibitor NU-7441 also inhibited the phosphorylation of B-Myb by GSK3β, suggesting that DNA-PK may also be involved in the phosphorylation of B-Myb in response to DSBs. This may be related to the recent observation that DNA-PK, in addition to its key role in DSB repair by non-homologous end-joining (NHEJ), also acts in concert with the MRN complex and ATM to regulate repair by homologous recombination (HR)^47^, or it may reflect a more general role of B-Myb in downstream signaling induced by DNA-DSB repair via HR and NHEJ.

Finally, we have demonstrated that mutation of the GSK3β phosphorylation sites of B-Myb interferes with the down-regulation of several pro-mitotic B-Myb target genes in response to DNA-damage (Fig. 8). Concurrently, the number of mitotic cells was increased. In addition we found that phosphorylated B-Myb associates with Lin9, which suggests that it is incorporated into the Myb-MuvB complex. Overall, this suggests that the DNA-damage induced, GSK3β-dependent phosphorylation of B-Myb decreases its transcriptional activity in the context of the Myb-MuvB complex, thereby contributing to the DNA-damage induced G2/M block. The sites phosphorylated by GSK3β are situated in the transactivation domain of B-Myb within the region that was previously shown to interact with p300^48^. It is therefore possible that the phosphorylation affects the binding of p300 or of other proteins interacting with this part of B-Myb, leading to a decreased transactivation potential of B-Myb. In summary, as illustrated in Fig. 8d our work suggests a novel function of B-Myb in cellular DNA-damage signaling that contributes to the damage induced G2/M-phase arrest. By showing that Cdk-dependent phosphorylation of B-Myb is suppressed by interaction of B-Myb with cyclin 7 at the onset of DNA-damage, Klein *et al*.^49^ have recently presented another mechanism by which B-Myb supports the G2/M-arrest. Both mechanisms may act hand-in-hand to convert the phosphorylation status of B-Myb from a Cdk- to a GSK3β-state.

## Methods

### Cells

Human MCF7, Hela and HEK293 cells were grown in DMEM supplemented with 10% fetal calf serum. HepG2 cells were grown in RPMI1640 medium containing 10% fetal calf serum and quail QT6 cells were grown in Iscove’s medium supplemented with 8% fetal calf serum and 2% chicken serum. Cells were irradiated with UV-C after removal of the culture medium and the tissue culture plate lid. If not noted otherwise BrdU (10 μM) was added to the growth medium 24–72 h before irradiation to sensitize the cells. After UV-C irradiation, the cells were allowed to recover in fresh medium for the indicated times. Kinase inhibitors were added 30 min before UV-C treatment at the indicated concentrations and re-added to fresh medium after irradiation.

### Micro-irradiation and Immunofluorescence

Micro-irradiation was performed as described previously^35^. Cells were grown on coverslips, washed with PBS, covered by porous polycarbonate filters (Isopore membrane, Millipore) and irradiated using a germicidal lamp. The cells were cultured further in fresh medium for the indicated times and fixed with 4% paraformaldehyde in PBS for 10 min. Cells were then permeabilized with PBS containing 0.2% NP40 for 15 min at room temperature and stained with the desired primary antibodies. Alexa488-coupled anti-mouse or Alexa546-coupled anti-rabbit IgG were used as secondary antibodies. Microscopic images were acquired with Leica TCS SL confocal system using the Leica confocal software (Leica Microsystems, Wetzlar, Germany). For co-localization analysis, the images were acquired sequentially and merged electronically using Adobe Photoshop software (Adobe Systems incorporated, San Jose, CA, USA).

### Quantification of mitotic cells

Cells were fixed with ethanol over night, washed twice with PBS (0.5% BSA) and incubated for 30 min with anti phospho-histone H3(Ser10) Alexa Fluor^®^ 488 conjugate, followed by washing with PBS (0.5% BSA). The cells were then incubated for 1 hour with propidium iodide (50 μg/ml containing 25 μg/ml RNaseA) and analyzed by flow cytometry.

### Lentiviral infections

Lentiviral vectors for Myc-tagged wild-type human B-Myb and B-Myb-Mut6 were generated by replacing the RFP coding region of pLVX-DsRed-Monomer-C1 (Clontech) by the coding sequence of wild-type B-Myb or B-Myb-Mut6. The resulting DNAs (pLVX-Puro Myc-BMybWT and pLVX-Puro Myc-BMybMut6) were cotransfected with the lentiviral packaging plasmids pMD2.G + pSPAX2 into HEK293T cells to generate infectious viral particles, followed by infection of HepG2 cells and puromycin selection to eliminate uninfected cells.

### Co-immunoprecipitation, GFP-trap and GST pull-down experiments

Cells were lysed in ELB buffer (50 mM Tris/HCl pH 7,5; 120 mM NaCl; 20 mM NaF; 1 mM EDTA; 6 mM EGTA; 15 mM sodium pyrophosphate; 1 mM phenylmethylsulfonyl fluoride; 0,2% NP-40 and a protease inhibitor mix containing Aprotinin, Leupeptin and Pepstatin). After incubation on ice for 15 min, lysates were centrifuged at 14 000 x g for 15 min, and the supernatant was used as total cell extract. Aliquots of the total cell extract were immunoprecipitated with the appropriate antibodies overnight at 4 °C. Protein-A Sepharose beads were then added and incubated further for 3 h at 4 °C under constant agitation. Immune complexes bound to the beads were collected by centrifugation, washed five times with lysis buffer and finally subjected to SDS-PAGE. Proteins were transferred to nitrocellulose membranes and stained with the appropriate antibodies. For GFP-trap experiments QT6 cells were transfected with expression vectors for GFP and GFP fusion proteins. Cells were lysed in ELB buffer 24 hours after transfection and the supernatant was used as total cell extract. Aliquots of cell extracts were then incubated with GFP-trap beads (Chromotec, München) for 3 h at 4 °C. Beads were washed 3 times with ELB buffer. Bound proteins and input samples were analyzed by western blotting. Expression and purification of GST-proteins and *in vitro* pull-down experiments were performed as described^18^.

### Phosphatase treatment

Cells were lysed in ELB buffer and B-Myb was immunoprecipitated with rabbit antiserum raised against the N-terminal part of B-Myb. Immunoprecipitates were transferred to phosphatase buffer (50 mM Tris/HCl, pH 8.5; 5 mM MgCl_2_) supplemented with 5 units FastAP (Thermo Scientific) and incubated at 37 °C for 1 h. Controls were incubated in the same buffer lacking phosphatase.

### Additional methods

These are presented in the Supplementary information.

## Additional Information

**How to cite this article**: Henrich, S. M. *et al*. Interplay with the Mre11-Rad50-Nbs1 complex and phosphorylation by GSK3b implicate human B-Myb in DNA-damage signalling. *Sci. Rep.* **7**, 41663; doi: 10.1038/srep41663 (2017).

**Publisher's note:** Springer Nature remains neutral with regard to jurisdictional claims in published maps and institutional affiliations.

## Supplementary Material

Supplemental Methods and Figures
