# Supplementary material for: Interplay with the Mre11-Rad50-Nbs1 complex and phosphorylation by GSK3β implicate human B-Myb in DNA-damage signaling
Source: Sci Rep. 2017 Jan 27;7:41663. doi: 10.1038/srep41663 (PMC5269693; doi:10.1038/srep41663)
Supplement: Supplemental Methods and Figures [file srep41663-s1.pdf]

Interplay with the Mre11-Rad50-Nbs1 complex and phosphorylation by GSK3b implicate human B-Myb in DNA-damage signaling

Sarah Marie Henrich, Clemens Usadel, Eugen Werwein, Kamila Burdova, Pavel Janscak, Stefano Ferrari, Daniel Hess and Karl-Heinz Klempnauer

Supplementary Methods

Antibodies. Staining for B-Myb was performed with the B-Myb LX015.1 mouse monoclonal antibody (Tavner et al., 2007), a kind gift by R. Watson. Immunoprecipitation of B-Myb was performed with rabbit antisera raised against the N-terminal (anti B-Myb#53) or the C-terminal (anti B-Myb Brian) part of B-Myb (Ziebold et al., 1997; Kamano et al.,1995). Rabbit antiserum against Lin9 was provided by S.Gaubatz. The following commercially available antibodies were used: mouse anti-Nbs1 (Genetex, GTX70224), mouse anti-Rad50 (Genetex, GTX70228), mouse anti-Mre11 (Genetex, GTX70212), rabbit anti-Nbs1 (Abcam, ab 32074), rabbit anti-phospho-Nbs1 (Genetex, GTX61779), rabbit anti-γH2AX (Genetex, GTX61796) and mouse anti-phospho-ATM (Biolegend, BLD-651201), mouse anti-GFP (Sigma-Aldrich, 7.1 + 13.1), mouse anti-β-actin (Sigma-Aldrich, AC-15), Alexa-Fluor®-488 conjugate of phospho-histoneH3(Ser10) (D2C8)XP®-rabbit mAb (Cell Signaling Technology, #3465).

Expression vectors and transfections. peGFP-B-Myb encodes a fusion proteins of eGFP and full-length human B-Myb. pYFP-B-Myb encodes a fusion protein of YFP with full-length mouse B-Myb. Truncated derivatives of human B-Myb encoding amino acids 1-207, 206-700, 206-509, 1-207 plus 485-700, 485-700, 1-509, 335-700, 207-335, 335-509 and 272-700 fused to eGFP were generated by cloning the corresponding parts of the B-Myb coding

region into peGFP-C1, 2 or 3, utilizing appropriate restriction sites. Expression vectors for GFP-B-Myb BM1, 2, 3 and 4 were generated by PCR and contain human B-Myb amino acids 190-260 (BM1), 260-355 (BM2), 355-435 (BM3) and 435-502 (BM4), fused C-terminally to GFP. Expression vectors for GFP-B-Myb fusion proteins containing human B-Myb amino acids 271-595 and 446-559 were generated by PCR with appropriate primers and cloning into peGFP-C1. The bacterial expression vector for His-tagged human B-Myb (amino acids 271-595) was generated by subcloning B-Myb sequences from plasmid peGFP-B-Myb(271-595) into bacterial expression vector pET28a+. The expression vector for GST/B-Myb(260-335) was obtained by PCR-amplifying the corresponding sequences from the coding region of human B-Myb and cloning it into pGEX-4T-1. B-Myb point mutants S454A, S480A, Mut3 (S282A,T286A,S287A) and Mut6 (S282A,T286A,S287A,S306A,S307A,S309A) were constructed by PCR using appropriate oligonucleotides to introduce the mutations. The expression vector for human Nbs1 (Psg5-Myc NBS1) was obtained from L.Pavic. Bacterial Nbs1 expression vectors GST-FHA (amino acids 1-118), GST-BRCT (amino acids 102-330) and GST-FHA/BRCT were generated by PCR using appropriate oligonucleotide primers and cloning between the EcoRI and XhoI (in case of GST-FHA and GST-FHA/BRCT) or BamHI and XhoI (in case of GST-BRCT) sites of pGex5X3. The expression vector for GST-Nbs1-CT (amino acids 572-754) has been described (Yuan et al., 2007). The expression vector for Flag-tagged human Rad50 was obtained from M.F.Lavin (Gatei et al., 2011). The expression vector for Flag-Mre11 was obtained from M. Meuth (Wen et al., 2008). Transient transfection of plasmid DNAs into QT6 fibroblasts was performed by calcium phosphate co-precipitation. An expression vector for HA-GSK3β was obtained from J. Woodgett (He et al., 1995). A GST-GSK3β expression vector was constructed by cloning the full-length coding region of GSK3β into pGEX-6p-2.

Repair Assays. Repair assays for homologous recombination (HR) and non-homologous end-joining (NHEJ) were performed as described (Pierce et al., 1999; Bennardo et al., 2008). The assay for HR, is based on HEK293T cells stably transfected with the pDR-GFP reporter

construct, which carries two mutant GFP genes, one of which contains a cutting site for the Sce1 nuclease. A DSB in this gene is repaired by HR between the two GFP mutant genes, resulting in the restoration of a functional GFP gene and the expression of GFP proteins. The NHEJ assay was carried out using the EJ5-GFP reporter (Bennardo et al., 2008), stably integrated in HEK293 cells. The EJ5-GFP contains a promoter that is separated from a GFP coding region by a puro gene which is flanked by two I-SceI sites. When the puro gene is removed by NHEJ repair of the two I-SceI-induced DSBs, the promoter is joined with the GFP coding region to restore a functional GFP gene. To measure HR and NHEJ repair efficiency the reporter cell lines were transfected with control or B-Myb siRNA and cultivated for 48 hours. The cells were then transfected with an expression vector for I-SceI (together with a boost of the corresponding siRNAs) and analyzed 54 hours later by flow cytometry to quantify the number of GFP-positive cells.

RNA interference. B-Myb expression was silenced with siRNA duplexes targeting the sequence 5'-GAAACAUGCUGCGUUUGUA-3' (B-Myb siRNA_4). siRNA targeting Renilla luciferase (5'-AAACAUGCAGAAAAUGCUG-3') was used as negative control. Cells were grown in 6 cm petri dishes to approximately 50–80% confluence and received 2.5 ml fresh growth medium before transfection. Then 100 nM siRNA was transfected using Lipofectamine® RNAiMAX Transfection Reagent (Invitrogen), according to manufacturers' protocols. Cells were harvested 24-72 h later, and the level of B-Myb knockdown was evaluated by western blotting.

Comet assay. UV-irradiated or etoposide-treated B-Myb knock-down or control HepG2 cells were washed with PBS and embedded in 0.5 % low melting point agarose (in PBS) on microscopic slides. The agarose was covered with cover-slip and left to solidify on ice for 5 min. After removing the cover-slip the slides were incubated for 2 to 24 h in lysis buffer (10 mM Tris-HCl, pH 10.0; 2.5 M NaCl, 100 mM EDTA, 1% TritonX-100). The sildes were then transferred to a cooled electrophoresis chamber and incubated on ice in electrophoresis

buffer (300 mM NaOH; 1mM EDTA) for 20 min before electrophoresis was started. After electrophoresis the slides were incubated in neutralization buffer (0.4 M Tris-HCl, pH 7.5) for 10 to 15 min at 4°C. After washing with distilled water, the cells were stained with propidium iodide and visualized in a confocal laser scanning microscope. 150 to 200 individual cells were analyzed for each condition, using the ImageJ-plugin „Open Comet".

Mass spectrometry analysis. The protein spots were excised from the gel, reduced with 10 mM TCEP, alkylated with 20 mM iodoacetamide and cleaved with 0.1 µg porcine sequencing grade trypsin (Promega) in 25 mM ammonium bicarbonate (pH 8.0) at 37°C for 16h. The extracted peptides were analyzed by capillary liquid chromatography tandem mass spectrometry with an EASY-nLC 1000 using the two-column set up (Thermo Scientific). The peptides were loaded in 0.1% formic acid, 2% acetonitrile in water onto a peptide trap (Acclaim PepMap 100, 75um x 2cm, C18, 3um, 100Å) at a constant pressure of 600 bar. Then they were separated, at a flow rate of 150 nl/min with a linear gradient of 2-6 % buffer B in buffer A in 3 minutes followed by an linear increase from 6 to 22 % in 40 minutes, 22-28 % in 9 min, 28-36 % in 8min, 36-80 % in 1 min and the column was finally washed for 12 min at 80% B (Buffer A: 0.1% formic acid, buffer B: 0.1% formic acid in acetonitrile) on a 50um x 15cm ES801 C18, 2um, 100Å column mounted on a DPV ion source (New Objective) connected to a Orbitrap Fusion (Thermo Scientific). The data were acquired using 120000 resolution for the peptide measurements in the Orbitrap and a top T (3s) method with a combination of consecutive CID and HCD fragmentation for each precursor and fragment measurement in the LTQ. Mascot Distiller 2.5 was used to combine the HCD and the CID spectra for individual precursors and MASCOT 2.5 searching swissprot version 2015_01 or a custom DB including the sequence GFP_B-Myb without the DBD domain was used to identify the phospho peptides. The enzyme specificity was set to trypsin allowing for up to three incomplete cleavage sites. Carbamidomethylation of cysteine (+57.0245) was set as a fixed modification, phosphorylation of serine, threonine and tyrosine (+79.9663 Da) oxidation of methionine (+15.9949 Da) and acetylation of protein N-termini (+42.0106 Da) were set as

variable modifications. Parent ion mass tolerance was set to 5 ppm and fragment ion mass tolerance to 0.6 Da. The results were validated with the program Scaffold Version 4.4 and ScaffoldPTM (Proteome Software, Portland, USA). Peptide identifications were accepted if they could be established at greater than 50.0 % probability as specified by the Peptide Prophet algorithm (Keller et. al, 2002; Nesvizhskii et al., 2003) and phosphorylation sited were accepted if they had a greater than 80% site probability as calculated with ScaffoldPTM. Relative quantification of the peptides was done with Progenesis LC-MS (Nonlinear Dynamics).

Real-time PCR. Total cellular RNA was isolated by TRIzol® (Ambion Life Technologies) extraction, following the instructions of the manufacturer. First strand cDNA synthesis was performed with a cDNA kit (SuperScript VILO Master Mix, Thermo Fisher Scientific). The cDNAs were analyzed by quantitative real-time PCR with the following primers. Cdc2: 5'-CATGGCTACCACTTGACCTGT-3' and 5'- AAGCCGGGATCTACCATACC-3', CycB1: 5'-CAGATGTTTCCATTGGGCTT-3' and 5'- TACCTATGCTGGTGCCAGTG-3', Plk1: 5'-GGCAACCTTTTCCTGAATGA-3' and 5'-TCCCACACAGGGTCCTTCTTC-3`, β-actin: 5'-CGTCCACCGCAAATGCTT-3' and 5'-GTTTTCTGCGCAAGTTAGGT-3'.

In vitro protein kinase assay. In vitro protein kinase assays using bacterially expressed GST-proteins were performed as follows. HA-tagged GSK3β was isolated by immunoprecipitation with anti-HA antibodies from QT6 cells transfected with an expression vector for HA-GSK3β. As control, an identical immunoprecipitation was performed with untransfected cells. Cells were lysed in ELB-buffer and immunoprecipitates were washed extensively with the same buffer. GST proteins were purified from bacterial extracts by binding to Glutathione sepharose beads, which were then mixed with protein-A sepharose beads carrying the immunoprecipitated HA-GSK3β and were incubated for 30 min at 37°C in kinase buffer (25 mM Tris-HCl, pH7.5, 10 mM $MgCl_2$, 5 mM beta-Glycerophosphat, 2 mM DTT, 100 mM Na_3VO_4, 50 μM ATP) containing 4 μCi of γ-^{32}P-ATP per reaction (Hartmann

Analytic, specific activity 3000 Ci/mmol). Reactions were stopped by adding SDS sample buffer. The reaction mixture was then subjected to 10 % SDS-PAGE and analyzed with a phosphor-image analyzer. Kinase assays with bacterially expressed GSK3β were performed similarly, except that GST-GSK3β, purified from bacterial extracts by binding to Glutathione sepharose beads, was used instead of the immunoprecipitated kinase.

Bennardo, N., Cheng, A., Huang, N. & Stark, J.M. Alternative-NHEJ is a mechanistically distinct pathway of mammalian chromosome break repair. *PLoS Genet.* **4**, e1000110 (2008).

Gatei, M. *et al.* ATM protein-dependent phosphorylation of Rad50 protein regulates DNA repair and cell cycle control. *J. Biol. Chem*. **286**, 31542-31556 (2011).

He, X., Saint-Jeannet, J.P., Woodgett, J.R., Varmus, H.E. & Dawid, I.B. Glycogen synthase kinase-3 and dorsoventral patterning in Xenopus embryos. *Nature* **374**, 617-622 (1995)

Kamano, H., Burk, B., Noben-Trauth, K. & Klempnauer, K.-H. Differential splicing of the mouse B-myb gene. *Oncogene* **11**, 2575-2582 (1995).

Keller, A., Nesvizhskii, A.I., Kolke, E. & Aebersold, R. Empirical statistical model to estimate the accuracy of peptide identifications made by MS/MS and database search. *Anal. Chem.* **74**, 5383-5392 (2002).

Nesvizhskii, A.I., Keller, A., Kolker, E. & Aebersold, R. A statistical model for identifying proteins by tandem mass spectrometry. *Anal. Chem.* **75** 4646-4658 (2003).

Pierce, A.J., Johnson, R.D., Thompson, L.H. & Jasin, M. XRCC3 promotes homology-directed repair of DNA damage in mammalian cells. *Genes Dev.* **13**, 2633–2638 (1999).

Tavner, F., Frampton, J. & Watson, R.J. Targeting an E2F site in the mouse genome prevents promoter silencing in quiescent and post-mitotic cells. *Oncogene* **26**, 2727-2735 (2007).

Wen, Q. *et al*. A mutant allele of MRE11 found in mismatch repair-deficient tumor cells suppresses the cellular response to DNA replication fork stress in a dominant negative manner. *Mol. Biol. Cell*. **4**, 1693-1705 (2008).

Yuan, Z., Zhang, X., Sengupta, N., Lane, W.S. & Seto, E. SIRT1 regulates the function of the Nijmegen breakage syndrome protein. *Mol. Cell* **27**, 149–162 (2007).

Ziebold, U., Bartsch, O., Marais, R., Ferrari, S. & Klempnauer, K.-H. Phosphorylation and activation of B-Myb by cyclin A-Cdk2. *Curr. Biol*. **7**, 253-260 (1997).

Supplementary Figure Legends

Supplementary Fig. 1. **B-Myb is associated with the MRN complex**. Extracts from untreated MCF7 cells were precipitated with antibodies against B-Myb DNA binding domain (anti B-Myb#53) (panel A) or the B-Myb C-terminal domain (anti B-Myb Brian) (panel B). As control, antiserum against an unrelated protein (GST) was used. In panel C, cell extracts were supplemented with 500 μg/ml ethidium bromide before immunoprecipitation. Immunoprecipitates and aliquots of the total cell extract (TCE) were immunoblotted using antibodies against Rad50, Nbs1, Mre11 and B-Myb.

Supplementary Fig. 2. **Visualization of DNA-damage foci after UVC-irradiation through micropore filters**. MCF7 cells grown for 72 h in BrdU-containing medium were irradiated with UVC light (30J/m2). After fixation the cells were analyzed by immunofluorescence microscopy with the indicated antisera.

Supplementary Fig. 3. **B-Myb accumulates at sites of DNA-damage in cells not pre-labeled with BrdU**. UV-microirradiated MCF7 cells not pre-incubated with BrdU were UVC-

irradiated (300J/m2) through micropore filters, fixed and analyzed by immunofluorescence microscopy with antibodies against B-Myb, pNbs1(Ser-343) and γH2AX.

Supplementary Fig. 4. **B-Myb is not essential for the repair of DNA double strand breaks. A,B.** GFP-based DNA repair assays. The NHEJ and HR repair substrates (EJ5SceGFP and DR-GFP) are illustrated schematically on the right. The panels on the left show the NHEJ (panel E) and HR (panel F) repair efficiencies in control and B-Myb knock-down cells.

Supplementary Fig. 5. **Comet assays. A,B**. HepG2 cells were transfected with control or B-Myb siRNA. 72 h later the cells were UV-irradiated (30J/m^2) and cultivated further for the indicated times. Non-irradiated cells (untreated) were used as control. Panel A shows representative pictures of cells subjected to the comet assay and panel B shows the quantification of the extent of DNA damage from a single experiment. Each column is derived from the analysis of 50 to 100 individual cells. Western blots showing B-Myb and β-actin expression in B-Myb knock-down and control cells are shown at the top. **C**. Control and B-Myb siRNA transfected cells were used without DNA-damage treatment (untreated) or were exposed to 2.6 μM etoposide for up to 180 min, as indicated. In addition, cells were treated with etoposide for 60 min, followed by further incubation for 180 min in the absence of etoposide. The cells were then analyzed by the comet assay as in panel B. **D**. Summary of comet assays derived from several independent experiments. The columns show the amount of DNA damage of control or B-Myb siRNA transfected HepG2 cells 60 min after UV irradiation or addition of etoposide. To facilitate the comparison of different experiments the amount of DNA damage in the control siRNA treated cells was set to 100 %.

Supplementary Fig. 6. **UV-induced mobility shifts of B-Myb in Hela and MCF7 cells**. The cells were prelabeled with BrdU for 72 hr, followed by UV-irradiation and western blotting of total cell extracts with B-Myb specific antibodies.

Supplementary Fig. 7. **Example spectra for a peptide with Ser282 and Ser287 phosphorylation.** The b- and y-ions are shown and the site analysis as calculated with MASCOT 2.5 is shown.

suppl. Fig. 1

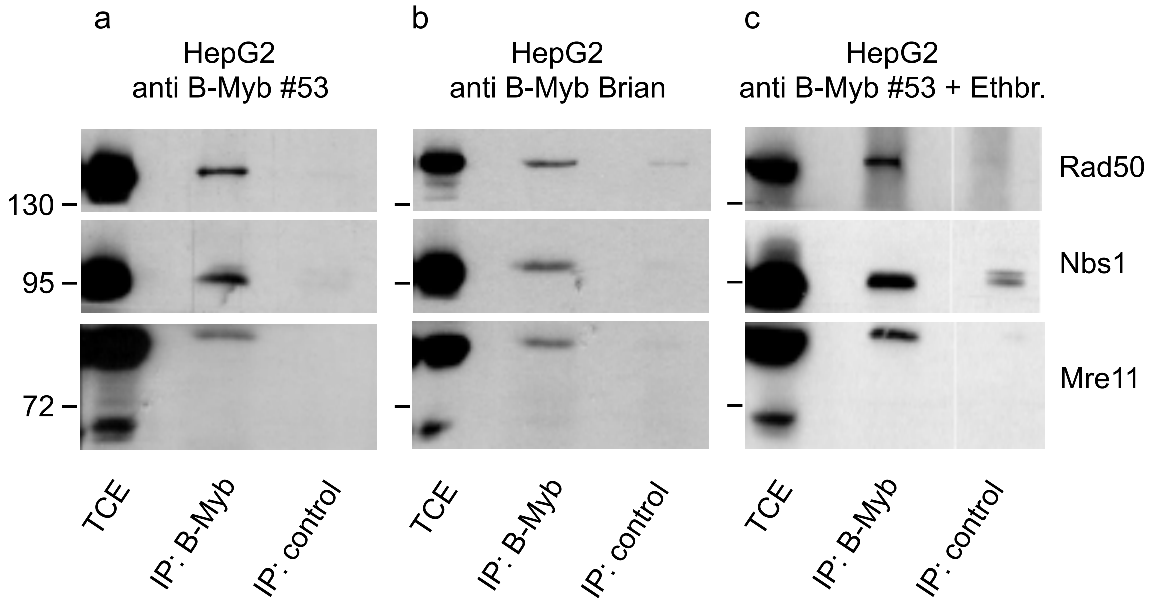
a
HepG2
anti B-Myb #53
b
HepG2
anti B-Myb Brian
c
HepG2
anti B-Myb #53 + Ethbr.
130
95
72
Rad50
Nbs1
Mre11
TCE
IP: B-Myb
IP: control
TCE
IP: B-Myb
IP: control
TCE
IP: B-Myb
IP: control

suppl. Fig. 2

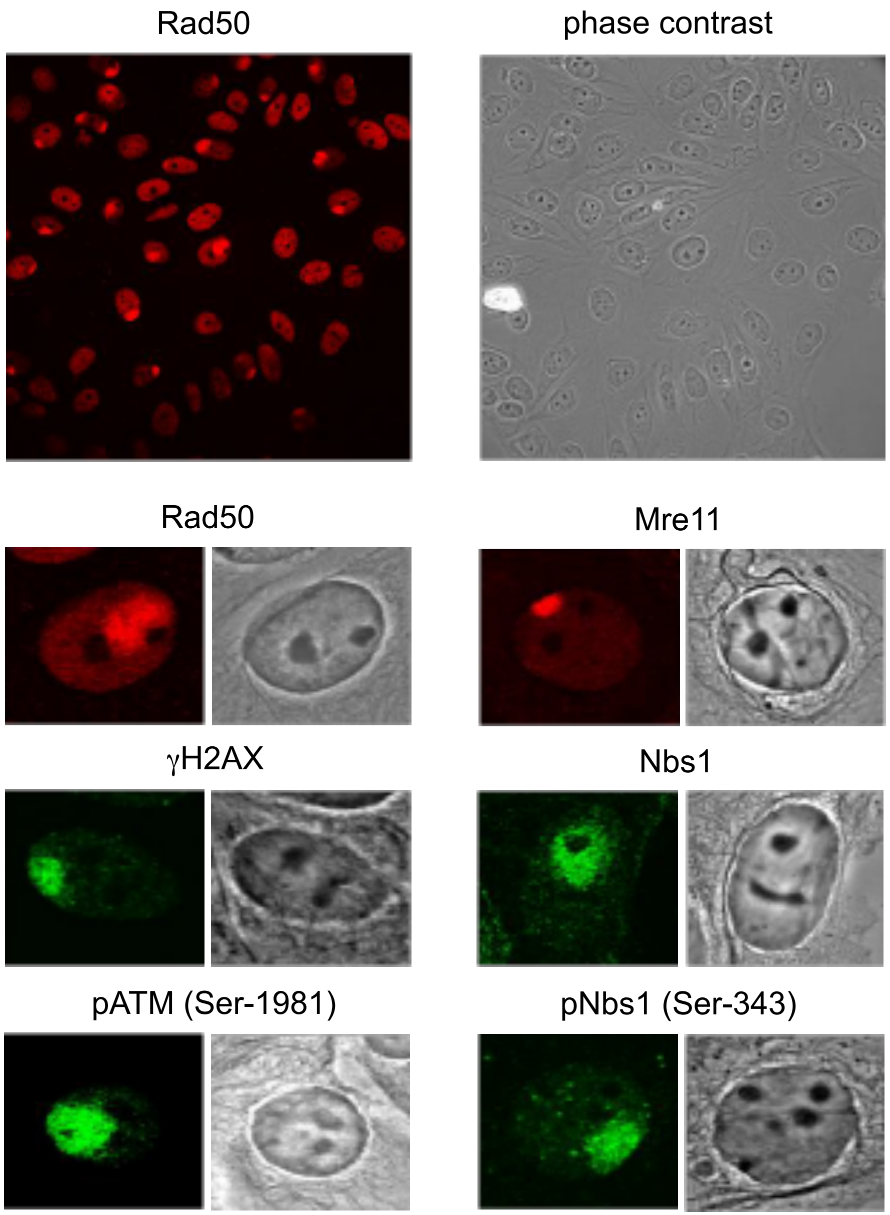
Rad50
phase contrast
Rad50
Mre11
γH2AX
Nbs1
pATM (Ser-1981)
pNbs1 (Ser-343)

suppl. Fig. 3

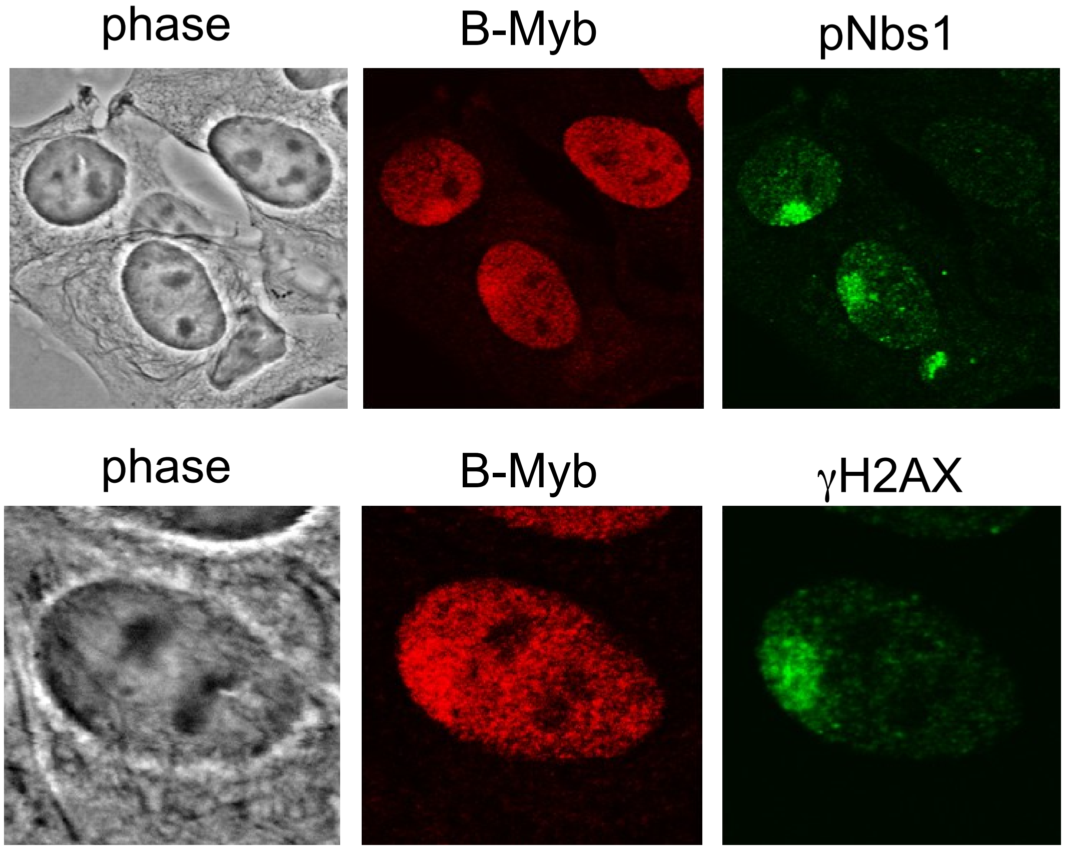

suppl. Fig. 4

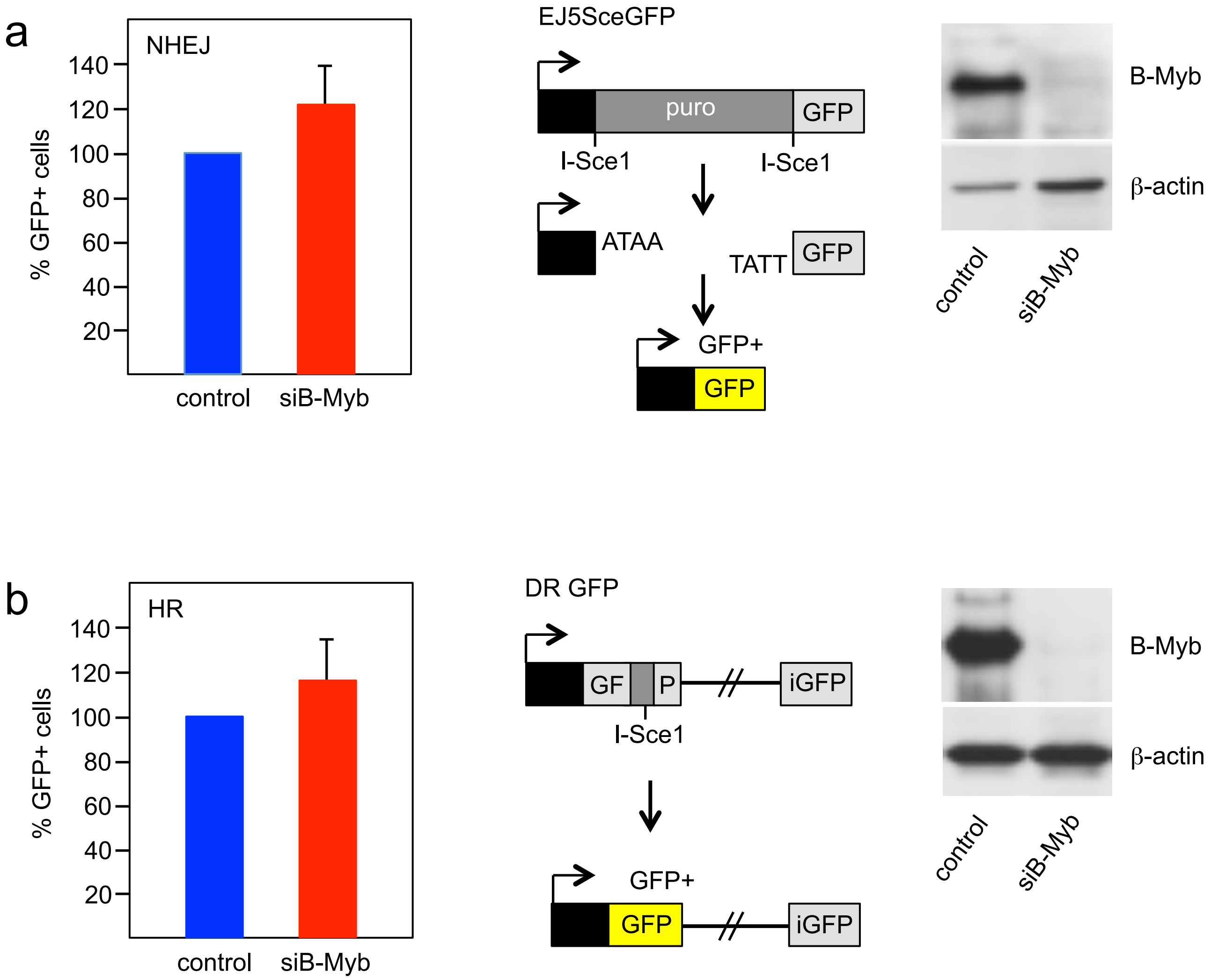
a
NHEJ
% GFP+ cells
140
120
100
80
60
40
20
control
siB-Myb
EJ5SceGFP
puro
GFP
I-Sce1
I-Sce1
ATAA
TATT
GFP
GFP+
GFP
B-Myb
β-actin
control
siB-Myb
b
HR
% GFP+ cells
140
120
100
80
60
40
20
control
siB-Myb
DR GFP
GF
P
iGFP
I-Sce1
GFP+
GFP
iGFP
B-Myb
β-actin
control
siB-Myb

suppl. Fig. 5

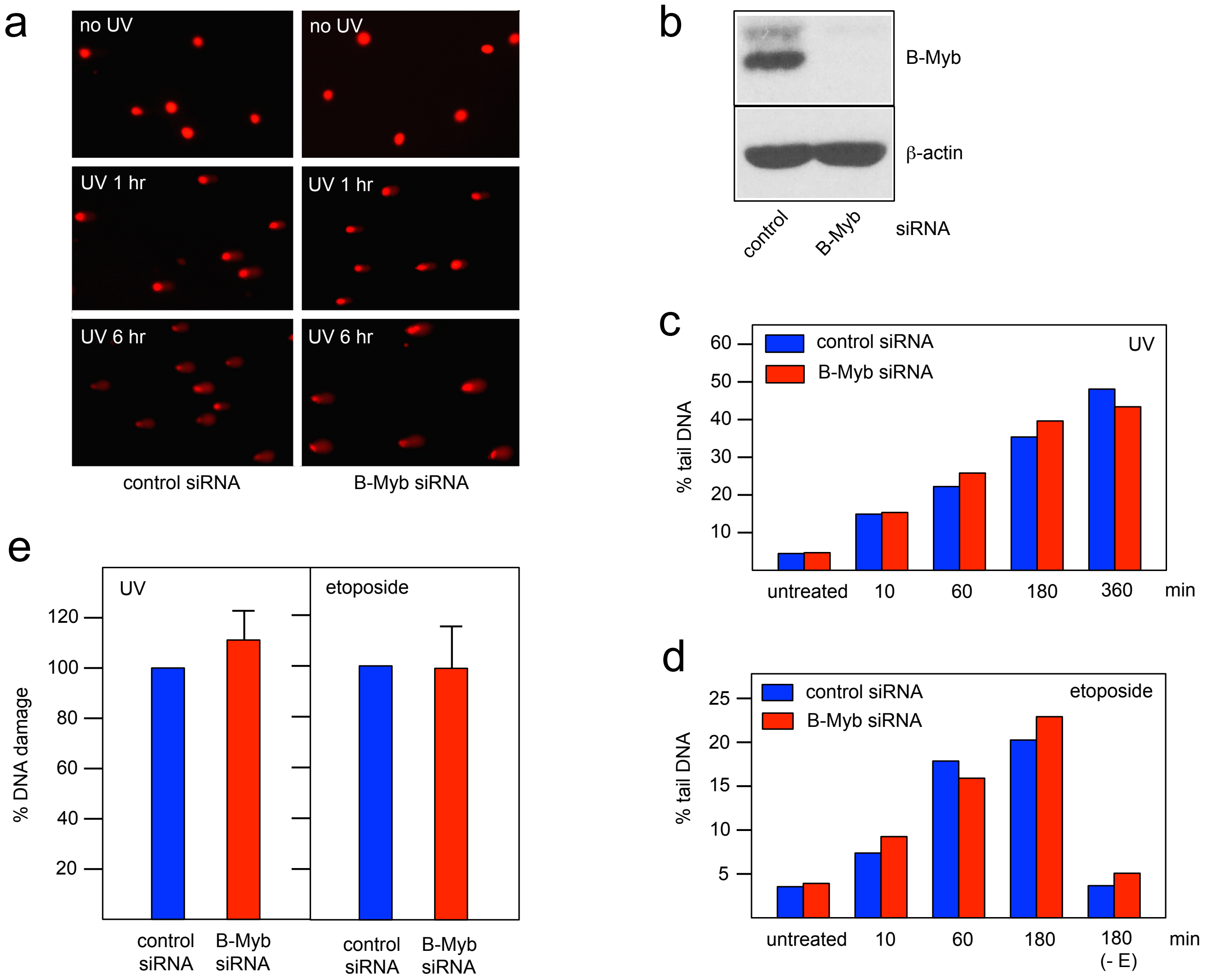
a
no UV
no UV
UV 1 hr
UV 1 hr
UV 6 hr
UV 6 hr
control siRNA
B-Myb siRNA
b
B-Myb
β-actin
control
B-Myb
siRNA
c
control siRNA
B-Myb siRNA
UV
% tail DNA
60
50
40
30
20
10
untreated
10
60
180
360
min
d
control siRNA
B-Myb siRNA
etoposide
% tail DNA
25
20
15
10
5
untreated
10
60
180
180 (- E)
min
e
UV
etoposide
% DNA damage
120
100
80
60
40
20
control siRNA
B-Myb siRNA
control siRNA
B-Myb siRNA

suppl. Fig. 6

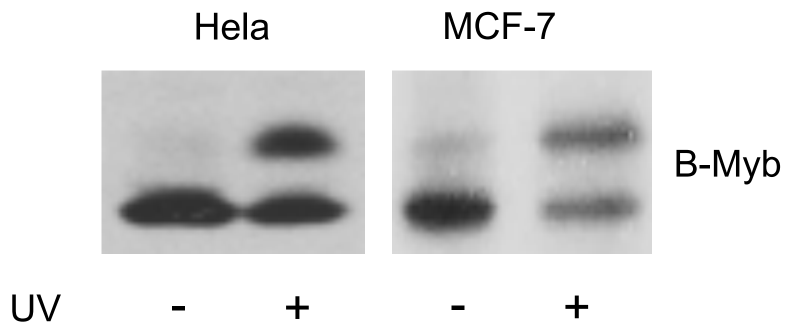

suppl. Fig. 7

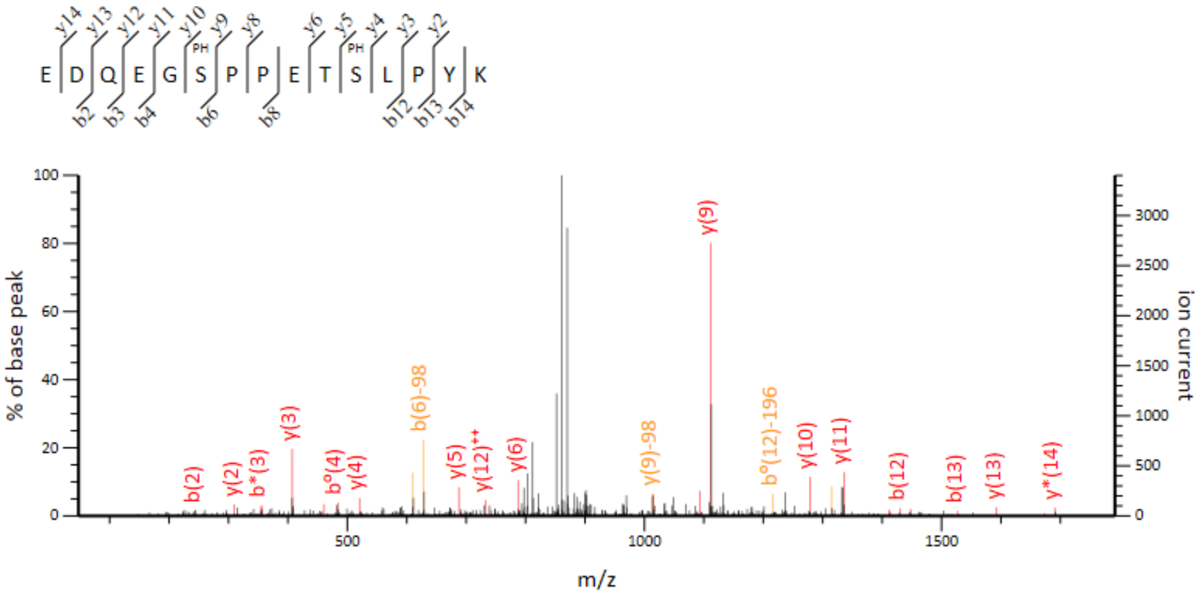

All matches to this query

Score	Mr(calc)	Delta	Sequence	Site Analysis
53.7	1835.6903	0.0013	EDQEGSPPETSLPYK	Phospho S6, S11 91.60%
42.9	1835.6903	0.0013	EDQEGSPPETSLPYK	Phospho S6, T10 7.71%
32.0	1835.6903	0.0013	EDQEGSPPETSLPYK	Phospho T10, S11 0.63%
30.9	1835.6903	0.0013	EDQEGSPPETSLPYK	
22.2	1835.6903	0.0013	EDQEGSPPETSLPYK	
18.3	1835.6903	0.0013	EDQEGSPPETSLPYK	
